# Activities of daily living as a longitudinal moderator of the effect of autonomic dysfunction on anxiety and depression of Parkinson's patients

**DOI:** 10.1002/brb3.2297

**Published:** 2021-08-01

**Authors:** Jing Cui, Yao Qin, Yuling Tian, Xiaoyan Ge, Hongjuan Han, Zongfang Yang, Hongmei Yu

**Affiliations:** ^1^ Department of Health Statistics School of Public Health Shanxi Medical University Taiyuan China; ^2^ Department of Neurology First Hospital of Shanxi Medical University Taiyuan China; ^3^ Shanxi Provincial Key Laboratory of Major Diseases Risk Assessment Taiyuan China

**Keywords:** activities of daily living, anxiety, depression, longitudinal mediation, Parkinson's disease

## Abstract

**Background:**

There is no clear time point for the onset of depression and anxiety in Parkinson's disease (PD), and their atypical physical symptoms often overlap with other nonmotor symptoms. Autonomic dysfunction usually appears earlier than motor symptoms, seriously impairing activities of daily living (ADL), even quality of life. Whether autonomic dysfunction can affect depression and anxiety in PD patients through ADL is still unclear.

**Methods:**

We conducted three progressive autoregressive mediation models to evaluate whether ADL may mediate the association between autonomic symptom burden, where the mediation chain with autonomic function as an independent variable, ADL as a mediating variable, and anxiety and depression as dependent variables. The ADL of PD patients were measured by the Scales for Outcomes in Parkinson's disease‐Autonomic (SCOPA‐AUT) and Modified Schwab and England ADL scale, respectively, and the status of depression and anxiety were measured by the Geriatric Depression Scale (GDS) and State‐Trait Anxiety Inventory (STAI).

**Results:**

There were 338 PD patients, including 220 males and 118 females. Demographic information, including age, gender, and education level, were not correlated with the depression and anxiety. Model III had the smallest AIC (AIC = 12,669.89), and the cross‐lagged relations were not statistically significant, so we selected Model II as the optimal model. In Model II, longitudinal autoregressive mediated effect and longitudinal mediated effect of autonomic dysfunction affecting anxiety and depression through ADL were not statistically significant, suggesting longitudinal changes of autonomic dysfunction were independent of anxiety and depression through ADL. Contemporaneous mediated effects of autonomic dysfunction affecting anxiety and depression through ADL were statistically significant, suggesting contemporaneous autonomic dysfunction may contribute to anxiety and depression through ADL.

**Conclusions:**

Targeted prevention and intervention measures for autonomic dysfunction and ADL should be taken to preserve and improve self‐perceived life satisfaction in the clinical practice and preventive health care of PD.

## Graphical abstract

Path graphs of autoregressive model I, II and III

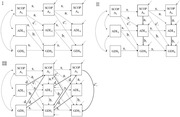

## 1 INTRODUCTION

Parkinson's disease (PD) is the most common movement disorder and represents the second most common neurological disorder, with its incidence and prevalence on the rise steadily with age (GBD 2015 Disease and Injury Incidence and Prevalence Collaborators, 2016 ). PD affects one to two people per 1000 at any one time, especially the elderly, causing a lot of burden to patients and their families, as well as to the entire society (Whetten‐Goldstein et al., 1997). It has been estimated that nearly 9 million people worldwide will suffer from PD by 2030 (Dorsey et al., 2007). The average life expectancy after diagnosis of PD is 7–15 years (Golbe & Leyton, 2018). So far, there are no disease‐modifying treatments, which can stop or delay the disease process or mortality. The main motor symptoms of PD patients are collectively referred to as “parkinsonism,” including bradykinesia, cogwheel rigidity, resting tremor, a slow shuffling gait, and imbalance (Pringsheim et al., 2013 ). Although PD is dominated by motor symptoms, a broad array of nonmotor symptoms (NMS) are probably overshadowed by more striking and frequent motor symptoms of PD (Sveinbjornsdottir, 2016), and it took to appreciate and measure the impact of NMS on PD till the 21st century (Martinez‐Martin et al., 2011). It is now evident that NMS not only occur across all motor stages of PD but also in premotor stages (Gallagher et al., 2010), such as autonomic dysfunction like constipation and urinary urgency, psychological symptoms like depression and anxiety, causing patients more trouble and earlier than their motor issues (Weintraub & Burn, 2011).

Depressive disorders are the most common condition among neuropsychiatric symptoms (Maiti et al., 2017), with a prevalence ranging from 40% to 50% (Marsh & Laura, 2013). Due to the overlap of PD depressive symptoms and nonmotor symptoms and the lack of clear criteria to evaluate and diagnose depression in PD patients, there is often insufficient intervention or delay (Blonder & Slevin, 2011). In addition to inherent emotional distress, depressive disorders have a negative impact on quality of life, cognition and function of PD patients. Unfortunately, depression is underrecognized and undertreated in clinical practice (Ravina et al., 2007). Studies have shown that depressive disorders can develop at any stage of the PD, predating the onset of motor symptoms by 4–6 years (Global Parkinson's Disease Survey (GPDS) Steering Committee, 2002). Anxiety is often comorbid with depression and also can occur independently (Kristine et al., 2012). Menza and colleagues reported that 92% of PD diagnosed with anxiety had depression, and 67% of PD patients with depression had anxiety (Menza et al., 1993). Anxiety and depression have become determinants of poorer quality of life, worse functional status and cognitive function of PD patients (Duncan et al., 2014). However, neurologists pay much attention to the impact of depression on patients (Pontone et al., 2011), but ignore anxiety (Chen & Marsh, 2014).

Autonomic dysfunction is present in virtually all PD patients from mild to life‐threatening (Abbott et al., 2003), with prevalence ranging from 14% to 80% (Berganzo et al., 2012). The natural history of autonomic nervous dysfunction in PD is still poorly unclear, such as swallowing, sleep disorders, cardiovascular dysregulation, and sexual dysfunction (Kaye et al., 2006). Gastrointestinal dysfunction causes impaired pharmacodynamics and deterioration of motor function, and urinary retention from bladder abnormalities causes septicemia and death (Palma & Kaufmann, 2018). Especially, orthostatic hypotension is present in some 50% of PD patients and results in increased debilitation and more frequent syncope, falls and fractures in the later stages of PD (Zesiewicz et al., 2003). These autonomic dysfunction symptoms in PD patients (Tomic et al., 2017) often appear earlier than motor symptoms, seriously impairing the quality of life (Merola et al., 2017; Müller et al., 2013). Aristide Merola proposed that worsening of autonomic dysfunction symptoms was associated with impairments in the activities of daily living (ADL), even after adjusting for disease duration, cognitive impairment, and motor severity (Merola et al., 2017). Miriam Sklerov reported longitudinal changes in autonomic dysfunction directly or indirectly affected ADL in early PD through the effect on depressive symptoms (Sklerov et al., 2020). Both subjective and objective measures of autonomic dysfunction in PD appear to have a negative impact on ADL (Merola et al., 2016; Sklerov et al., 2020). The impact of autonomic nervous dysfunction on ADL needs to be further investigated.

Due to the insidious onset of PD, there is no clear time point for the onset of depression, anxiety and autonomic dysfunction, and their atypical physical symptoms often overlap with other comorbide NMS. Inadequate health care and resources and side effects of dopaminergic medication make these assessments in PD patients often complex (Gallagher & Schrag, 2012). Mediation analysis, widely applied in psychological and behavioral research, provides an effective way to reveal the “black box” of underlying processes (Dodhia, 2005; Lorenzo et al., 2013). Most efforts of mediation have been based on cross‐sectional data, ignoring any consideration of time sequence (Shrout & Bolger, 2002). Maxwell pointed out that cross‐sectional mediation analysis typically generated substantially biased estimates of longitudinal mediated effects, and implied the existence of a substantial indirect effect (Maxwell et al., 2011). However, the relationship between depression, anxiety and autonomic dysfunction in PD patients may vary with the development of the disease. Longitudinal mediation analysis can explore a more complete possible interaction mechanism, allowing researchers to investigate how the process develops over time (Shrout, 2011).

In this study, we hypothesized that anxiety and depression were affected by autonomic dysfunction through ADL of PD patients. With this aim, we used longitudinal mediation analysis to explore the impact and relationship between depression, anxiety and autonomic dysfunction, to provide suggestions for alleviating and preventing the depression and anxiety of PD.

## 2 MATERIALS AND METHODS

### 2.1 Participants

The data for this study were from the Parkinson's Progression Markers Initiative (PPMI), an observational, international, multicenter study designed to establish a longitudinal biomarker—defined early PD cohort, to improve understanding of the etiology and course of PD (Simuni et al., 2018). For the PPMI study, written informed consent was obtained for all participants and the study protocol was approved by the institutional review board at each participating center, before protocol‐specific procedures were performed. The dataset was downloaded on July 2, 2018, including 338 PD patients who were followed for three years with an interval of one year. Please refer to the website (www.ppmi‐info.org) for detailed study design, inclusion and exclusion criteria, standard protocols, registration, and consent procedures.

General information included gender, age, educational level, family history, and disease duration. The Geriatric Depression Scale (GDS) (Ertan et al., 2005), the State‐Trait Anxiety Inventory (STAI), and Scales for Outcomes in Parkinson's disease‐Autonomic (SCOPA‐AUT) (Visser et al., 2004) were used to assess depression, anxiety, and autonomic symptom burden of PD patients, respectively. STAI included the STAI state subscale (S‐anxiety) and STAI trait subscale (T‐anxiety) (Newham et al., 2012). The Modified Schwab & England ADL Scale (S & E ADL) was used to assess the disability in ADL and severity of dyskinesias, with higher scores indicating better function (Eusebi et al., 2018; Marek et al., 2018).

### 2.2 Statistical analysis

An autoregressive mediation model was performed to evaluate whether ADL may mediate the association between autonomic symptom burden, as measured by the SCOPA‐AUT, and depression and anxiety, as measured by the GDS and STAI. The autoregressive mediation model included two parts: path analysis and mediation analysis. Path analysis was used to reflect the stability of clinical measurements during follow‐ups. For mediated effect analysis, due to the difference between *p*‐value point estimation and CI test estimation, there may be conflicting situations where *p* > 0.05 but CI did not contain zero. *p*‐Value point estimation was based on the Sobel test, while CI test estimation was derived from the bias‐corrected bootstrap test (Fritz & Mackinnon, 2007). At present, researchers believed that the statistical power of CI was greater, so we adopted CI to test the statistical significance of the mediation. Statistical significance was obtained whenever the 95% CI fell outside the value of zero. As an exploratory study, Akaike information criterion (AIC) and Bayesian information criterion (BIC) provided an attractive basis for model selection. We used SPSS25.0 to perform basic statistical description of demographic information at baseline and clinical assessments of follow‐ups, and explored associations between clinical assessments by Spearman correlation coefficients. We used Mplus software to build a longitudinal mediation model. *p* Values of less than 0.05 were considered significant.

## 3 RESULTS

### 3.1 Demographic and clinical measurements

There were 338 PD patients, including 220 males and 118 females. The age range of patients was 33.5–84.9 years. The age interval of onset and disease duration was 25.4–83.0 years and 0.4–37.0 months, respectively. Demographic information at baseline and correlations with depression and anxiety of the first year are summarized in Table S1. Since demographic information, such as age, gender, and education level, were not correlated with depression and anxiety, so they were not included in the subsequent longitudinal mediation models. Repeated measurements of depression, anxiety, autonomic dysfunction and ADL in the three follow‐ups and their correlations are shown in Tables S2 and S3, respectively. Autonomic nerve function and ADL of PD patients declined as the disease progressed, and the degree of depression and anxiety increased. The three repeated measurements of depression, anxiety, autonomic dysfunction and ADL were correlated with each other, especially ADL was negatively correlated with anxiety, depression and autonomic dysfunction. So, independent simple mediation analyses at three times may lead to biased estimates of mediated effects.

### 3.2 Autoregressive mediation Model I

#### 3.2.1 Path analysis of Model I

**FIGURE 1:**
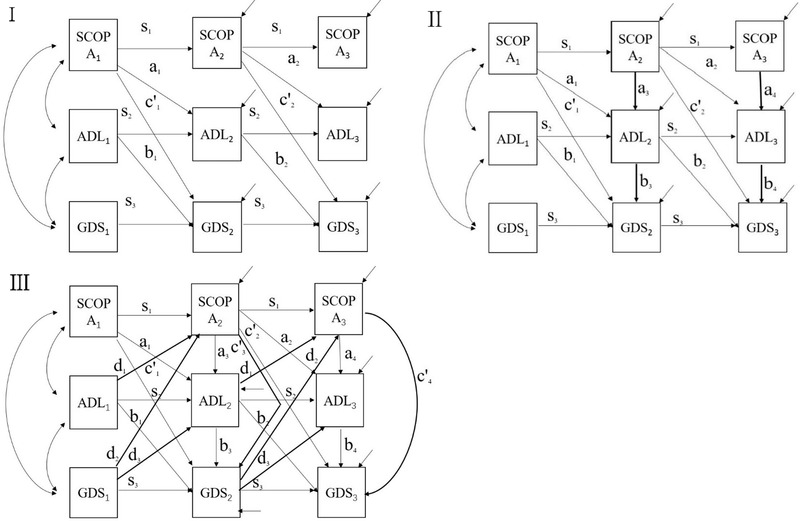
Path graphs of autoregressive Models I, II, and III

Depression and anxiety were used as dependent variables for path analysis (Table 1). The path graph can be seen in Figure 1(I). As shown in Table 1, the path coefficients *s*_1_, *s*_2_, and *s*_3_ of anxiety (STAI_1 _→ STAI_2_, STAI_2 _→ STAI_3_), depression (GDS_1 _→ GDS_2_, GDS_2 _→ GDS_3_), ADL (ADL_1 _→ ADL_2_, ADL_2 _→ ADL_3_) and autonomic dysfunction (SCOPA_1 _→ SCOPA_2_, SCOPA_2 _→ SCOPA_3_) during three repeated measurements, were statistically significant, indicating that clinical measurements were stable during the follow‐up period. On the path SCOPA_1 _→ STAI_3_, the path coefficients of the dependent variable anxiety STAI_2_ and the independent variable autonomic dysfunction SCOPA_1_ ($c_{1}^{\prime }=0.056$, *p* = 0.194) and the mediator ADL_1_ (*b*_1 _= 0.011, *p* = 0.772) were not statistically significant. The path SCOPA_2 _→ STAI_3_ was statistically insignificant ($c_{2}^{\prime }=0.026$, *p* = 0.497). Similarly, on the path SCOPA_1 _→ GDS_3_, the path coefficients of the dependent variable depression GDS_2_ and the independent variable autonomic dysfunction SCOPA_1_ ($c_{1}^{\prime }=0.056$, *p* = 0.194) and the mediator ADL_1_ (*b*_1 _= 0.011, *p* = 0.772) were not statistically significant. The path SCOPA_2 _→ GDS_3_ was statistically insignificant ($c_{2}^{\prime }=0.026$, *p* = 0.497).

**TABLE 1:** Path and mediation analysis of autoregressive mediation Model I (β (95% CI))

Path effect		Mediated effect	
SCOPA1 → SCOPA2	0.766 (0.712,0.808)***	Autonomic dysfunction → Anxiety	
SCOPA2 → SCOPA3	0.774 (0.720,0.819)***	*a*_1_ *b*_1_	−0.003 (−0.026,0.018)
SCOPA1 → ADL2	−0.159 (−0.243,−0.063)**	*a*_2_ *b*_2_	0.014 (0.002,0.039)
ADL1 → ADL2	0.549 (0.454,0.634)***	*a*_1_ *b*_2_	0.027 (0.005,0.067)
SCOPA2 → ADL3	−0.085 (−0.170,−0.003)*	Total indirect effect	0.039 (0.005,0.091)
ADL2 → ADL3	0.600 (0.503,0.688)***	AIC	12,689.929
ADL1 → STAI2	0.011 (−0.063,0.081)	BIC	12,793.151
SCOPA1 → STAI2	0.056 (−0.022,0.147)	Autonomic dysfunction → Depression	
STAI1 → STAI2	0.715 (0.626,0.785)***	*a*_1_ *b*_1_	−0.006 (−0.053,0.036)
ADL2 → STAI3	−0.115 (−0.212,−0.025)*	*a*_2_ *b*_2_	0.028 (0.004,0.078)
SCOPA2 → STAI3	0.026 (−0.051,0.099)	*a*_1_ *b*_2_	0.055 (0.010,0.134)
STAI2 → STAI3	0.734 (0.649,0.799)***	Total indirect effect	0.077 (0.010,0.183)
ADL1 → GDS2	0.011 (−0.170,0.227)	AIC	13,627.064
SCOPA1 → GDS2	0.056 (−0.075,0.405)	BIC	13,730.286
GDS1 → GDS2	0.715 (0.608,0.805)***		
ADL2 → GDS3	−0.115 (−0.480,−0.055)*		
SCOPA2 → GDS3	0.026 (−0.137,0.287)		
GDS2 → GDS3	0.734 (0.666,0.827)***		

Abbreviations: ADL, activities of daily living; SE, standard error; AIC, Akaike information criterion; BIC, Bayesian information criterion; GDS, Geriatric Depression Scale; SCOPA‐AUT, Scales for Outcomes in Parkinson's disease‐Autonomic; STAI, State‐Trait Anxiety Inventory.

* *p* < .05;

** *p* < .01;

*** *p* < .001.

#### 3.2.2 Mediation analysis of Model I

The mediated effect results of autoregressive Model I are shown in Table 1, where AIC values of mediation chains SCOPA_1 _→ STAI_3_ and SCOPA_1 _→ GDS_3_ were 12,689.929 and 13,627.064, respectively. On the mediation chains of autonomic dysfunction affecting anxiety through ADL during three years of follow‐ups, mediated effect *a*_1_ *b*_1_ was insignificant. The *p*‐values of the longitudinal autoregressive mediated effect *a*_2_ *b*_2_ and the longitudinal mediated effect *a*_1_ *b*_2_ were both greater than 0.05, but the Bootstrap 95% confidence interval (CI) did not contain 0. Due to the greater power of the Bootstrap bias correction than the Sobel test, it was considered that *a*_2_ *b*_2_ and *a*_1_ *b*_2_ were both significant. Similarly, the total indirect effect was significant, indicating that autonomic dysfunction during the follow‐up affected ADL and then affected anxiety. Similar to the above‐mentioned mediation chain, the mediated effects *a*_2_ *b*_2_, *a*_1_ *b*_2_ and total indirect effect of autonomic dysfunction on depression through ADL were statistically significant. It showed that the autonomic dysfunction of PD patients would affect depression by affecting ADL during the follow‐up process.

### 3.3 Autoregressive mediation Model II

#### 3.3.1 Path analysis of Model II

Figure 1(II) shows the path graph of autoregressive mediation Model II, adding *a*_3_, *b*_3_, *a*_4_, and *b*_4_ contemporaneous paths in the same period based on autoregressive Model I, to test contemporaneous mediated effects *a*_3_ *b*_3_ and *a*_3_ *b*_4_. As shown in Table 2, the path analysis results of Model II are different from Model I.

**TABLE 2:** Path and mediation analysis of autoregressive mediation Model II

Path effect		Mediated effect	
SCOPA1 → SCOPA2	0.766 (0.712,0.808)***	Autonomic dysfunction → Anxiety	
SCOPA2 → SCOPA3	0.774 (0.720,0.819)***	*a*_1_ *b*_1_	−0.003 (−0.033,0.026)
SCOPA1 → ADL2	−0.017 (−0.178,0.146)	*a*_2_ *b*_2_	0.001 (−0.006,0.023)
ADL1 → ADL2	0.546 (0.453,0.631)***	*a*_3_ *b*_3_	0.038 (0.003,0.106)
SCOPA2 → ADL2	−0.187 (−0.355,−0.017)*	*a*_4_ *b*_4_	0.048 (0.009,0.114)
SCOPA2 → ADL3	0.038 (−0.092,0.179)	*a*_1_ *b*_2_	0.000 (−0.020,0.010)
ADL2 → ADL3	0.596 (0.500,0.685)***	Total indirect effect	0.084 (0.015,0.184)
SCOPA3 → ADL3	−0.158 (−0.312,−0.018)*	AIC	12,677.427
ADL1 → STAI2	0.09 (0.005,0.168)*	BIC	12,784.472
SCOPA1 → STAI2	0.038 (−0.039,0.127)	Autonomic dysfunction → Depression	
STAI1 → STAI2	0.708 (0.618,0.78)***	*a*_1_ *b*_1_	−0.005 (−0.066,0.051)
ADL2 → STAI2	−0.146 (−0.242,−0.051)**	*a*_2_ *b*_2_	0.002 (−0.012,0.045)
ADL2 → STAI3	0.018 (−0.079,0.107)	*a*_3_ *b*_3_	0.077 (0.007,0.213)
SCOPA2 → STAI3	0.006 (−0.070,0.077)	*a*_4_ *b*_4_	0.096 (0.015,0.227)
STAI2 → STAI3	0.725 (0.637,0.792)***	*a*_1_ *b*_2_	−0.001 (−0.039,0.019)
ADL3 → STAI3	−0.228 (−0.332,−0.135)***	Total indirect effect	0.169 (0.030,0.367)
ADL1 → GDS2	0.09 (0.005,0.168)*	AIC	13,614.562
SCOPA1 → GDS2	0.038 (−0.039,0.127)	BIC	13,721.607
STAI1 → STAI2	0.708 (0.618,0.78)***		
ADL2 → STAI2	−0.146 (−0.242,−0.051)**		
ADL2 → STAI3	0.018 (−0.079,0.107)		
SCOPA2 → STAI3	0.006 (−0.070,0.077)		
STAI2 → STAI3	0.725 (0.637,0.792)***		
ADL3 → STAI3	−0.228 (−0.332,−0.135)***		
ADL1 → GDS2	0.09 (0.005,0.168)*		
SCOPA1 → GDS2	0.038 (−0.039,0.127)		
GDS1 → GDS2	0.708 (0.618,0.78)***		
ADL2 → GDS2	−0.146 (−0.242,−0.051)**		
ADL2 → GDS3	0.018 (−0.079,0.107)		
SCOPA2 → GDS3	0.006 (−0.170,0.077)		
GDS2 → GDS3	0.725 (0.637,0.792)***		
ADL3 → GDS3	−0.228 (−0.332,−0.135)***		

Abbreviations: ADL, activities of daily living; SE, standard error; AIC, Akaike information criterion; BIC, Bayesian information criterion; GDS, Geriatric Depression Scale; SCOPA‐AUT, Scales for Outcomes in Parkinson's disease‐Autonomic; STAI, State‐Trait Anxiety Inventory.

* *p* < .05;

** *p* < .01;

*** *p* < .001.

On the mediation chains of autonomic dysfunction affecting anxiety through ADL, path ADL_1 _→ STAI_2_ (*b*_1 _= 0.090, *p* = 0.029) was significant, but the mediated effect *a*_1_ *b*_1_ of path SCOPA_1 _→ ADL_2_ (*a*_1 _= −0.017, *p* = 0.841) and path SCOPA_2 _→ STAI_3_ ($c_{2}^{\prime }=0.006$, *p* = 0.880) remained insignificant. At the same time, the newly added paths were all significant, including SCOPA_2 _→ ADL_2_ (*a*_3 _= −0.187, *p* = 0.031), SCOPA_3 _→ ADL_3_ (*a*_4 _= −0.158, *p* = 0.034), ADL_2 _→ STAI_2_ (*b*_3 _= −0.146, *p* = 0.002), ADL_3 _→ STAI_3_ (*b*_4 _= −0.228, *p* < 0.001), suggesting that contemporaneous mediated effects *a*_3_ *b*_3_ and *a*_4_ *b*_4_ of autonomic dysfunction affecting anxiety through ADL were significant. Similarly, the path ADL_1 _→ GDS_2_ (*b*_1 _= 0.090, *p* = 0.029) became significant and the path coefficient was equal to ADL_1 _→ STAI_2_. The path ADL_2 _→ GDS_3_ (*b*_2 _= 0.018, *p* = 0.706) lost statistical significance, and the path SCOPA_2 _→ GDS_3_ ($c_{2}^{\prime }=0.006$, *p* = 0.880) was still not significant. The newly added paths were all significant, including ADL_2 _→ GDS_2_ (*b*_3 _= −0.146, *p* = 0.003), ADL_3 _→ GDS_3_ (*b*_4 _= −0.228, *p* < 0.001), suggesting that contemporaneous mediated effects *a*_3_ *b*_3_ and *a*_4_ *b*_4_ of autonomic dysfunction affecting depression through ADL were significant.

#### 3.3.2 Mediation analysis of Model II

As shown in Table 2, where AIC values of mediation chains SCOPA_1 _→ STAI_3_ and SCOPA_1 _→ GDS_3_ were 12,677.427 and 13,614.562, respectively. On the mediation chains of autonomic dysfunction affecting anxiety through ADL, longitudinal autoregressive mediated effect *a*_1_ *b*_1_ (*p* = 0.085, 95%CI (−0.033, 0.026)) and *a*_2_ *b*_2_ (*p* = 0.862, 95%CI (−0.006, 0.023)) were not significant. The contemporaneous mediated effects *a*_3_ *b*_3_, *a*_4_ *b*_4_ and total indirect effect were all significant. Mediation chain of depression were similar to anxiety, where longitudinal autoregressive mediated effects *a*_1_ *b*_1_ (*p* = 0.853, 95%CI (−0.066, 0.051)) and *a*_2_ *b*_2_ (*p* = 0.862, 95%CI (−0.012, 0.045)) were not significant, and mediated effects *a*_3_ *b*_3_, *a*_4_ *b*_4_ and total indirect effect were significant.

### 3.4 Autoregressive mediation Model III

#### 3.4.1 Path analysis of Model III

Figure 1(III) shows the path graph of autoregressive mediation Model III. Compared with Model II, the independent variable model added the dependent variables at the previous time point; the mediator model added the dependent variables at the previous time point; dependent variable model added the independent variables at the same time point. Model III can judge whether the cross‐lagged relations existed, that is, whether *d*_1_, *d*_2_, and *d*_3_ were significant. It can be seen from Table 3 that path coefficients between repeated measurements of each clinical evaluation on the mediation chain were still significant. Identical to results of Model II, paths SCOPA_1 _→ ADL_2_ (*a*_1 _= −0.011, *p* = 0.895), SCOPA_2 _→ ADL_3_ (*a*_2 _= 0.047, *p* = 0.496), SCOPA_1 _→ STAI_2_ ($c_{1}^{\prime }=-0.084$, *p* = 0.149), SCOPA_2 _→ STAI_3_ ($c_{2}^{\prime }=-0.065$, *p* = 0.271), ADL_2 _→ STAI_3_ (*b*_3 _= 0.015, *p* = 0.748) remained insignificant. Similar to anxiety as dependent variable, paths SCOPA_1 _→ GDS_2_ ($c_{1}^{\prime }=-0.084$, *p* = 0.149), SCOPA_2 _→ GDS_3_ ($c_{2}^{\prime }=-0.065$, *p* = 0.271), ADL_2 _→ GDS_3_ (*b*_3 _= 0.015, *p* = 0.748) remained insignificant. In addition, the newly added paths were not significant, indicating the absence of the cross‐lagged relations newly included in Model III.

**TABLE 3:** Path analysis of autoregressive mediation Model III

SCOPA1 → STAI3		SCOPA1 → GDS3	
Path	β (95% CI)	Path	β (95% CI)
SCOPA1 → SCOPA2	0.744 (0.687,0.791)***	ADL1 → SCOPA2	−0.020 (−0.071,0.024)
ADL1 → SCOPA2	−0.020 (−0.071,0.024)	GDS1 → SCOPA2	0.054 (−0.001,0.109)
STAI1 → SCOPA2	0.054 (−0.001,0.109)	ADL2 → SCOPA3	−0.022 (−0.079,0.025)
SCOPA2 → SCOPA3	0.747 (0.679,0.803)***	GDS2 → SCOPA3	0.049 (−0.001,0.099)
ADL2 → SCOPA3	−0.022 (−0.079,0.025)	GDS1 → ADL2	−0.028 (−0.094,0.04)
STAI2 → SCOPA3	0.049 (−0.001,0.099)	GDS2 → ADL3	−0.028 (−0.091,0.039)
SCOPA1 → ADL2	−0.011 (−0.170,0.152)	SCOPA1 → GDS2	−0.084 (−0.194,0.032)
SCOPA2 → ADL2	−0.180 (−0.352,−0.014)*	SCOPA2 → GDS2	0.171 (0.054,0.286)**
ADL1 → ADL2	0.543 (0.449,0.629)***	ADL1 → GDS2	0.082 (−0.002,0.161)*
STAI1 → ADL2	−0.028 (−0.094,0.04)	ADL2 → GDS2	−0.127 (−0.219,−0.037)**
SCOPA2 → ADL3	0.047 (−0.085,0.189)	GDS1 → GDS2	0.693 (0.599,0.767)***
SCOPA3 → ADL3	−0.158 (−0.310,−0.018)*	SCOPA2 → GDS3	−0.065 (−0.186,0.048)
ADL2 → ADL3	0.590 (0.497,0.679)***	SCOPA3 → GDS3	0.094 (−0.015,0.217)
STAI2 → ADL3	−0.028 (−0.091,0.039)	ADL2 → GDS3	0.015 (−0.079,0.102)
SCOPA1 → STAI2	−0.084 (−0.194,0.032)	ADL3 → GDS3	−0.216 (−0.318,−0.127)***
SCOPA2 → STAI2	0.171 (0.054,0.286)**	GDS2 → GDS3	0.716 (0.628,0.786)***
ADL1 → STAI2	0.082 (−0.002,0.161)*		
ADL2 → STAI2	−0.127 (−0.219,−0.037)**		
STAI1 → STAI2	0.693 (0.599,0.767)***		
SCOPA2 → STAI3	−0.065 (−0.186,0.048)		
SCOPA3 → STAI3	0.094 (−0.015,0.217)		
ADL2 → STAI3	0.015 (−0.079,0.102)		
ADL3 → STAI3	−0.216 (−0.318,−0.127)***		
STAI2 → STAI3	0.716 (0.628,0.786)***		

Abbreviations: ADL, activities of daily living; GDS, Geriatric Depression Scale; SCOPA‐AUT, scales for outcomes in Parkinson's disease‐autonomic; SE, standard error; STAI, State‐Trait Anxiety Inventory.

* *p* <  .05;

** *p* < .01;

*** *p* < .001.

#### 3.4.2 Mediation analysis of Model III

As shown in Table 4, where AIC values of mediation chains SCOPA_1 _→ STAI_3_ and SCOPA_1 _→ GDS_3_ were 12,669.894 and 13,607.029, respectively. On the mediation chains of autonomic dysfunction affecting anxiety through ADL, contemporaneous mediated effects (*a*_3_ *b*_3 _= 0.032, 95%CI (0.003, 0.091); *a*_4_ *b*_4 _= 0.046, 95%CI (0.008, 0.108) and total indirect effect = 0.078, 95%CI (0.015, 0.172)) were all significant. Similarly, on the mediation chains of autonomic dysfunction affecting depression through ADL, contemporaneous mediated effects (*a*_3_ *b*_3 _= 0.065, 95%CI (0.005, 0.182); *a*_4_ *b*_4 _= 0.092, 95%CI (0.016, 0.215) and total indirect effect = 0.156, 95%CI (0.030, 0.344)) were all significant. It proved that autonomic dysfunction of PD patients in this study did affect anxiety and depression through ADL. Other mediated effects on these two mediation chains were not significant.

**TABLE 4:** Mediation analysis of autoregressive mediation Model III

Autonomic dysfunction → Anxiety	β (95% CI)	Autonomic dysfunction → Depression	β (95% CI)
a_1_b_1_	−0.002 (−0.031,0.024)	*a*_1_ *b*_1_	−0.003 (−0.062,0.048)
a_2_b_2_	0.001 (−0.006,0.022)	*a*_2_ *b*_2_	0.002 (−0.013,0.044)
a_3_b_3_	0.032 (0.003,0.091)	*a*_3_ *b*_3_	0.065 (0.005,0.182)
a_4_b_4_	0.046 (0.008,0.108)	*a*_4_ *b*_4_	0.092 (0.016,0.215)
a_1_b_2_	0.000 (−0.018,0.011)	*a*_1_ *b*_2_	−0.001 (−0.035,0.021)
Total indirect effect	0.078 (0.015,0.172)*	Total indirect effect	0.156 (0.030,0.344)*
AIC	12,669.894	AIC	13,607.029
BIC	12,796.055	BIC	13,733.190

*Note*: Depression and anxiety were dependent variables, respectively. And GDS in the path diagram can be replaced by STAI. The number after each variable represented the follow‐up time.

Abbreviations: ADL, activities of daily living; SE, standard error; AIC, Akaike information criterion; BIC, Bayesian information criterion; GDS, Geriatric Depression Scale; SCOPA‐AUT, Scales for Outcomes in Parkinson's disease‐Autonomic; STAI, State‐Trait Anxiety Inventory..

* *p* < .05;

** *p* < .01;

*** *p* < .001.

## 4 DISCUSSION

At present, PD cannot be cured, mainly to improve spiritual life and life quality. Therefore, it is necessary to effectively prevent depression and anxiety in PD patients (Schrag & Taddei, 2017). In this study, autonomic dysfunction, ADL, depression and anxiety were included in the longitudinal mediation analysis to explore their interaction mechanism in the course of PD, and provided a theory basis for the prevention and treatment of anxiety and depression in PD patients.

### 4.1 Demographic factors about anxiety and depression

Some studies argued anxiety and depression were associated with different demographic factors. Negre‐Pages found female PD patients under 62 were more likely to experience depression and anxiety, with more severe Parkinsonism and comorbidities (Nègre‐Pagès et al., 2010). It may be because female PD patients have less social support and greater psychological pressure (Pavon et al., 2010). Cui's work found that disease duration and education level were correlated to the anxiety and depression of PD patients (Cui et al., 2017). However, some studies pointed out that there was no correlation between the gender, age, education level and anxiety and depression of PD patients (Hongyang, 2017; Lijuan et al., 2009). Due to differences in scales of anxiety and depression, and the composition of patients, there was no evidence of correlations between demographic factors and anxiety and depression of PD patients in our study. Further researches are needed to understand these findings.

### 4.2 Autoregressive mediation model

For the mediation chain with anxiety and depression as dependent variables in the autoregressive mediation Model I, longitudinal autoregressive mediated effect and total mediated effect were both significant, indicating that autonomic dysfunction of PD affected ADL and then affected anxiety and depression. Compared with Model I, after adding contemporaneous clinical measurements of Model II, newly added path coefficients of path analysis were all significant, indicating contemporaneous mediated effects of autonomic dysfunction can affect anxiety and depression through ADL. However, mediated effect analysis found autoregressive mediated effect lost significance, and contemporaneous mediated effect and total indirect effect were significant. In summary, the interaction mechanism of autonomic dysfunction affecting anxiety and depression through ADL was somewhat different from Model I. Due to lower AIC and BIC values of Model II than Model I, it was believed that Model II is more realistic (Burnham & Anderson, 2004). Mackinnon pointed out that cross‐lagged relations cannot be observed in the cross‐sectional study, and it is more reasonable to consider them in the mediation analysis (Mackinnon, 2008). Compared with Model II in this study, cross‐lagged relations added in Model III were not significant, indicating that there were no cross‐lagged relations or not reaching a significant level. In addition, the AIC of Model III was the smallest, but BIC was higher than Model II. Therefore, it was believed that Model II of three autoregressive mediation models involved in this study performed better.

Previous studies have found that autonomic dysfunction existed at the initial diagnosis of PD, and it gradually worsened with disease progression (Verbaan et al., 2007). Autonomic dysfunction has previously been implicated in depression of PD patients (Sagna et al., 2014). Moreover, PD patients often have symptoms of anxiety and depression at the same time. Depression and anxiety in PD can occur at any stage of disease including pre‐motor phase in the same patient, probably because these two symptoms share a common pathophysiological mechanism (Dissanayaka et al., 2010). To sum up, this study confirmed the hypothesis proposed in the introduction: the autonomic dysfunction of PD patients would affect anxiety and depression through ADL, and interaction mechanism between them existed certain difference with disease progression. This finding indicated that anxiety and depression should not be treated independently of autonomic symptoms in PD patients.

There were still some shortcomings in this study. First, some important clinical markers were not included, such as the cognitive function, cerebrospinal fluid and neroimaging. Second, there may be heterogeneity in PD patients, and different studies should be given for different types of populations. In future studies, more clinical markers, such as motor severity, measured by MDS‐Unified Parkinson's Disease Rating Scale (MDS‐UPDRS III and IV), levodopa equivalent dose and intake of antidepressive drugs, should be included as much as possible to improve the mediation chain, and perform longitudinal mediation analysis after potential category analysis of PD patients, to deepen the understanding of the anxiety and depression mechanism.

This study found that autonomic dysfunction of PD patients had longitudinal mediated effect on the occurrence and aggravation of anxiety and depression through ADL. Targeted prevention and intervention measures for autonomic dysfunction and ADL should be taken to preserve and improve self‐perceived life satisfaction of PD patients in the clinical practice and preventive health care of PD.

## CONFLICT OF INTEREST

The authors declare no conflict of interest.

## AUTHOR CONTRIBUTIONS

Jing Cui, Yuling Tian and Zongfang Yang have made substantial contributions to conception and design, or acquisition of data, or analysis and interpretation of data. Jing Cui, Yao Qin and Xiaoyan Ge have been involved in drafting the manuscript or revising it critically for important intellectual content. Hongmei Yu and Hongjuan Han have given final approval of the version to be published.

## ETHICAL STATEMENT

For the PPMI study, written informed consent was obtained for all participants and the study protocol was approved by the institutional review board at each participating center, before protocol‐specific procedures were performed.

## Supporting information

SUPPORTING INFORMATION

## Data Availability

Data used in this paper were obtained from the Parkinson's Progression Markers Initiative (PPMI) database. Data used in the paper could be derived in a public repository to share.

## References

[brb32297-bib-0001] Abbott, R. D., Ross, G. W., White, L. R., Sanderson, W. T., Burchfiel, C. M., Kashon, M., & Petrovitch, H. (2003). Environmental, life‐style, and physical precursors of clinical Parkinson's disease: Recent findings from the Honolulu‐Asia Aging Study. Journal of Neurology, 250(3 Suppl.). 10.1007/s00415-003-1306-7 14579122

[brb32297-bib-0002] Berganzo, K., Tijero, B., Somme, J. H., Llorens, V., Sánchez‐Manso, J. C., Low, D., & Lezcano, E. (2012). SCOPA‐AUT scale in different parkinsonisms and its correlation with (123) I‐MIBG cardiac scintigraphy. Parkinsonism & Related Disorders, 250(1). 10.1007/s00415-003-1306-7 21908227

[brb32297-bib-0003] Blonder, L. X., & Slevin, J. T. (2011). Emotional dysfunction in Parkinson's disease. Behavioural Neurology, 24(3), 201–217. 10.3233/ben-2011-0329 21876260 PMC3177157

[brb32297-bib-0004] Burnham, K. P., & Anderson, D. R. (2004). Multimodel inference: Understanding AIC and BIC in model selection. Sociological Methods & Research, 33(2), 261–304. 10.1177/0049124104268644.

[brb32297-bib-0005] Chen, J. J., & Marsh, L. (2014). Anxiety in Parkinson's disease: Identification and management. Therapeutic Advances in Neurology Disorders, 7(1), 52–59. 10.1177/1756285613495723. PMC3886380 24409202

[brb32297-bib-0006] Cui, S. S., Du, J. J., Fu, R., Lin, Y. Q., Huang, P., He, Y. C., & Chen, S. D. (2017). Prevalence and risk factors for depression and anxiety in Chinese patients with Parkinson disease. BMC Geriatrics, 17(1), 270. 10.1186/s12877-017-0666-2. 29166864 PMC5700465

[brb32297-bib-0007] Dissanayaka, N. N. W., Sellbach, A., Matheson, S., O'Sullivan, J. D., & Mellick, G. D. (2010). Anxiety disorders in Parkinson's disease: Prevalence and risk factors. Movement Disorders Official Journal of the Movement Disorder Society, 25(7), 838–845. 10.1002/mds.22833. 20461800

[brb32297-bib-0008] Dodhia, R. M. (2005). A review of applied multiple regression/correlation analysis for the behavioral sciences (3rd ed.). Journal of Educational & Behavioral Statistics, 30(2), 227–229. 10.3102/10769986030002227.

[brb32297-bib-0009] Dorsey, E. R., Constantinescu, R., Thompson, J. P., Biglan, K. M., Holloway, R. G., Kieburtz, K., & Siderowf, A. (2007). Projected number of people with Parkinson disease in the most populous nations, 2005 through 2030. Neurology, 68(5), 384–386. 10.1212/01.wnl.0000247740.47667.03. 17082464

[brb32297-bib-0010] Duncan, G. W., Khoo, T. K., Yarnall, A. J., O'Brien, J. T., Coleman, S. Y., Brooks, D. J., Barker, R. A., & Burn, D. J. (2014). Health‐related quality of life in early Parkinson's disease: The impact of nonmotor symptoms. Movement Disorders, 29(2), 195–202. 10.1002/mds.25664. 24123307

[brb32297-bib-0011] Ertan, F. S., Ertan, T., Kızıltan, G., & Uyguçgil, H. (2005). Reliability and validity of the Geriatric Depression Scale in depression in Parkinson's disease. Journal of Neurology Neurosurgery & Psychiatry, 76(10), 1445. 10.1136/jnnp.2004.057984. PMC1739374 16170093

[brb32297-bib-0012] Eusebi, P., Romoli, M., Paolini‐Paoletti, F., Tambasco, N., & Parnetti, L. (2018). Risk factors of levodopa‐induced dyskinesia in Parkinson's disease: Results from the PPMI cohort. npj Parkinson's Disease, 4(1). 10.1038/s41531-018-0069-x. PMC6240081 30480086

[brb32297-bib-0013] Fritz, M. S., & Mackinnon, D. P. (2007). Required sample size to detect the mediated effect. Psychological Science, 18(3), 233–239. 10.1111/j.1467-9280.2007.01882.x. 17444920 PMC2843527

[brb32297-bib-0014] Gallagher, D. A., Lees, A. J., & Schrag, A. (2010). What are the most important nonmotor symptoms in patients with Parkinson's disease and are we missing them? Movement Disorders, 25(15), 2493–2500. 20922807 10.1002/mds.23394

[brb32297-bib-0015] Gallagher, D. A., & Schrag, A. (2012). Psychosis, apathy, depression and anxiety in Parkinson's disease. Neurobiology of Disease, 46(3), 581–589. 10.1016/j.nbd.2011.12.041. 22245219

[brb32297-bib-0016] GBD 2015 Disease and Injury Incidence and Prevalence Collaborators . (2016). Global, regional, and national incidence, prevalence, and years lived with disability for 310 diseases and injuries, 1990–2015: A systematic analysis for the Global Burden of Disease Study 2015. Lancet, 388(10053), 1545–1602. 10.1016/s0140-6736(16)31678-6. 27733282 PMC5055577

[brb32297-bib-0017] Global Parkinson's Disease Survey (GPDS) Steering Committee . (2002). Factors impacting on quality of life in Parkinson's disease: Results from an international survey. Movement Disorders, 17(1), 60–67. 10.1002/mds.10010. 11835440

[brb32297-bib-0018] Golbe, L. I., & Leyton, C. E. (2018). Life expectancy in Parkinson disease. Neurology, 91. 10.1212/WNL.000000000000656. 30381371

[brb32297-bib-0019] Hongyang, S. (2017). Clinical study of autonomic nerve dysfunction in patients with Parkinson's disease. Jilin University.

[brb32297-bib-0020] Kaye, J., Gage, H., Kimber, A., Storey, L., & Trend, P. (2006). Excess burden of constipation in Parkinson's disease: A pilot study. Movement Disorders, 21(8), 1270–1273. 10.1002/mds.20942. 16700046

[brb32297-bib-0021] Kristine, H., & Cronin‐Golomb, A. (2012). Impact of anxiety on quality of life in Parkinson's disease. Parkinsons Disease, 2012, 1–8, 10.1155/2012/640707. PMC3236448 22191074

[brb32297-bib-0022] Li Juan, L., Qiao Wei, L., Shao, H., & Yao, B. (2009). Incidence and related factors of depression and anxiety associated with Parkinson's disease. Guangdong Medicine, 30(002), 266–268.

[brb32297-bib-0023] Lorenzo, R., Rino, B., & Daniela, Z. (2013). Mediation analysis in epidemiology: Methods, interpretation and bias. International Journal of Epidemiology, 42(5), 1511–1519. 10.1093/ije/dyt127. 24019424

[brb32297-bib-0024] Mackinnon, D. P. (2008). Introduction to statistical mediation analysis. Introduction to Probability & Statistics for Engineers & Scientists, 36(279), 1–8.

[brb32297-bib-0025] Maiti, P., Manna, J., & Dunbar, G. L. (2017). Current understanding of the molecular mechanisms in Parkinson's disease: Targets for potential treatments. Translational Neurodegeneration, 6(1), 28. 10.1186/s40035-017-0099-z. 29090092 PMC5655877

[brb32297-bib-0026] Marek, K., Chowdhury, S., Siderowf, A., Lasch, S., Coffey, C. S., Caspell‐Garcia, C., & Trojanowski, J. Q. (2018). The Parkinson's progression markers initiative (PPMI) – Establishing a PD biomarker cohort. Annals of Clinical and Translational Neurology, 5, 1460–1477. 10.1002/acn3.644. 30564614 PMC6292383

[brb32297-bib-0027] Marsh, L. (2013). Depression and Parkinson's disease: Current knowledge. Current Neurology & Neuroscience Reports, 13(12), 1–9. 10.1016/bs.irn.2017.05.024. PMC4878671 24190780

[brb32297-bib-0028] Martinez‐Martin, P., Rodriguez‐Blazquez, C., Kurtis, M. M., Chaudhuri, K. R., & Group, O. B. O. V. (2011). The impact of non‐motor symptoms on health‐related quality of life of patients with Parkinson's disease. Movement Disorders, 26(3), 399–406. 10.1002/mds.23462. 21264941

[brb32297-bib-0029] Maxwell, S. E., Cole, D. A., & Mitchell, M. A. (2011). Bias in cross‐sectional analyses of longitudinal mediation: Partial and complete mediation under an autoregressive model. Multivariate Behavioral Research, 46(5), 816–841. 10.1080/00273171.2011.606716. 26736047

[brb32297-bib-0030] Menza, M. A., Robertson‐Hoffman, D. E., & Bonapace, A. S. (1993). Parkinson's disease and anxiety: Comorbidity with depression. Biological Psychiatry, 34(7), 465. 10.1016/0006-3223(93)90237-8. 8268331

[brb32297-bib-0031] Merola, A., Romagnolo, A., Rosso, M., Lopez‐Castellanos, J. R., Wissel, B. D., Larkin, S., & Lopiano, L. (2016). Orthostatic hypotension in Parkinson's disease: Does it matter if asymptomatic? Parkinsonism & Related Disorders, 65. 10.1016/j.parkreldis.2016.09.013. 27641792

[brb32297-bib-0032] Merola, A., Romagnolo, A., Rosso, M., Suri, R., Berndt, Z., Maule, S., & Espay, A. J. (2017). Autonomic dysfunction in Parkinson's disease: A prospective cohort study. Movement Disorders, 33, 391–397. 10.1002/mds.27268. 29278286

[brb32297-bib-0033] Müller, B., Assmus, J., Herlofson, K., Larsen, J. P., & Tysnes, O. B. (2013). Importance of motor vs. non‐motor symptoms for health‐related quality of life in early Parkinson's disease. Parkinsonism & Related Disorders, 19(11). 10.1016/j.parkreldis.2013.07.010. 23916654

[brb32297-bib-0034] Nègre‐Pagès, L., Grandjean, H., Lapeyre‐Mestre, M., Montastruc, J. L., Fourrier, A., Lépine, J. P., & Group, O. B. O. S. (2010). Anxious and depressive symptoms in Parkinson's disease: The French cross‐sectionnal DoPaMiP study. Movement Disorders, 25(2), 157–166. 10.1002/mds.22760. 19950403

[brb32297-bib-0035] Newham, J. J., Westwood, M., Aplin, J. D., & Wittkowski, A. (2012). State‐trait anxiety inventory (STAI) scores during pregnancy following intervention with complementary therapies. Journal of Affective Disorders, 142(1‐3), 22–30. 10.1016/j.jad.2012.04.027. 22959685

[brb32297-bib-0036] Palma, J. A., & Kaufmann, H. (2018). Treatment of autonomic dysfunction in Parkinson disease and other synucleinopathies. Movement Disorders, 33(3), 372–390. 10.1002/mds.27344. 29508455 PMC5844369

[brb32297-bib-0037] Pavon, J. M., Whitson, H. E., & Okun, M. S. (2010). Parkinson's disease in women: A call for improved clinical studies and for comparative effectiveness research. Maturitas, 65(4), 352–358. 10.1016/j.maturitas.2010.01.001. 20117891 PMC2875870

[brb32297-bib-0038] Pontone, G. M., Williams, J. R., Anderson, K. E., Chase, G., Goldstein, S. R., Grill, S., & Margolis, R. L. (2011). Anxiety and self‐perceived health status in Parkinson's disease. Parkinsonism & Related Disorders, 17(4), 249–254. 10.1016/j.parkreldis.2011.01.005. 21292531 PMC3081400

[brb32297-bib-0039] Pringsheim, T., Jette, N., Frolkis, A., & Steeves, T. D. L. (2013). The prevalence of Parkinson disease: A systematic review and meta‐analysis (P03.067). Movement Disorders 10.1002/mds.25945. 24976103

[brb32297-bib-0040] Ravina, B., Camicioli, R., Como, P. G., Marsh, L., Jankovic, J., Weintraub, D., & Elm, J. (2007). The impact of depressive symptoms in early Parkinson disease. Neurology, 69(4), 342–347. 10.1212/01.wnl.0000268695.63392.10. 17581943 PMC2031220

[brb32297-bib-0041] Sagna, A., Gallo, J. J., & Pontone, G. M. (2014). Systematic review of factors associated with depression and anxiety disorders among older adults with Parkinson's disease. Parkinsonism & Related Disorders, 20(7), 708–715. 10.1016/j.parkreldis.2014.03.020. 24780824 PMC4648277

[brb32297-bib-0042] Schrag, A., & Taddei, R. N. (2017). Depression and anxiety in Parkinson's disease. International Review of Neurobiology, 133, 623–655. 10.1016/bs.irn.2017.05.024. 28802935

[brb32297-bib-0043] Shrout, P. E. (2011). Commentary: Mediation analysis, causal process, and cross‐sectional data. Multivariate Behav Res, 46(5), 852–860. 10.1080/00273171.2011.606718 26736049

[brb32297-bib-0044] Shrout, P. E., & Bolger, N. (2002). Mediation in experimental and nonexperimental studies: New procedures and recommendations. Psychol Methods, 46(4), 852–860. 10.1080/00273171.2011.606718. 12530702

[brb32297-bib-0045] Simuni, T., Siderowf, A., Lasch, S., Coffey, C. S., Caspell‐Garcia, C., Jennings, D., Tanner, C. M., Trojanowski, J. Q., Shaw, L. M., Seibyl, J., Schuff, N., Singleton, A., Kieburtz, K., Toga, A. W., Mollenhauer, B., Galasko, D., Chahine, L. M., Weintraub, D., Foroud, T., Tosun, D., … Parkinson's Progression Marker Initiative , (2018). Longitudinal change of clinical and biological measures in early Parkinson's disease: Parkinson's progression markers initiative cohort. Movement Disorders Official Journal of the Movement Disorder Society, 33(5), 771–782. 10.1002/mds.27361. 29572948 PMC6001458

[brb32297-bib-0046] Sklerov, M., Shih, C. H., Browner, N., Palma, J. A., & Dayan, E. (2020). Longitudinal change in autonomic symptoms predicts activities of daily living and depression in Parkinson's disease. Clinical Autonomic Research, 30(2008), 223–230. 10.1007/s10286-020-00672-7. 32078091

[brb32297-bib-0047] Sveinbjornsdottir, S. (2016). The clinical symptoms of Parkinson's disease. Journal of Neurochemistry, 139, 318–324. 10.1111/jnc.13691. 27401947

[brb32297-bib-0048] Tomic, S., Rajkovaca, I., Pekic, V., Salha, T., & Misevic, S. (2017). Impact of autonomic dysfunctions on the quality of life in Parkinson's disease patients. Acta Neurologica Belgica, 117(1), 207–211. 10.1007/s13760-016-0739-6. 28028676

[brb32297-bib-0049] Verbaan, D., Marinus, J., Visser, M., Rooden, S. V., Stiggelbout, A. M., & Hilten, J. V. (2007). Patient‐reported autonomic symptoms in Parkinson disease. Neurology, 69(4), 333–341. 10.1212/01.wnl.0000266593.50534.e8. 17646625

[brb32297-bib-0050] Visser, M., Marinus, J., Stiggelbout, A. M., & Hilten, J. J. V. (2004). Assessment of autonomic dysfunction in Parkinson's disease: The SCOPA‐AUT. Movement Disorders, 19(11), 1306–1312. 10.1002/mds.20153. 15390007

[brb32297-bib-0051] Weintraub, D., & Burn, D. J. (2011). Parkinson's disease: The quintessential neuropsychiatric disorder. Movement Disorders, 26(6), 1022–1031. 10.1002/mds.23664. 21626547 PMC3513835

[brb32297-bib-0052] Whetten‐Goldstein, K., Sloan, F., Kulas, E., Cutson, T., & Schenkman, M. (1997). The burden of Parkinson's disease on society, family, and the individual. Journal of the American Geriatrics Society, 45(7), 844–849. 10.1111/j.1532-5415.1997.tb01512.x. 9215336

[brb32297-bib-0053] Zesiewicz, T. A., Baker, M. J., Wahba, M., & Hauser, R. A. (2003). Autonomic nervous system dysfunction in Parkinson's disease. Current Treatment Options in Neurology, 5(2), 149–160. 10.1007/s11940-003-0005-0. 12628063

