# Supplementary material for: Activities of daily living as a longitudinal moderator of the effect of autonomic dysfunction on anxiety and depression of Parkinson's patients
Source: Brain Behav. 2021 Aug 1;11(8):e2297. doi: 10.1002/brb3.2297 (PMC8413789; doi:10.1002/brb3.2297)
Supplement: Supplementary file 1 — SUPPORTING INFORMATION [file BRB3-11-e2297-s001.docx]

**Supplementary Table 1. Demographics information about participants (N=338)**

| Variable | Group | N(%) | Depression | | Anxiety | |
| --- | --- | --- | --- | --- | --- | --- |
|  |  |  | r | p | r | p |
| Gender | Male | 220(65.09) | 0.005 | 0.933 | -0.029 | 0.602 |
|  | Female | 118(34.91) |  |  |  |  |
| Age (year) | <56 | 99(29.29) | 0.056 | 0.322 | 0.056 | 0.322 |
|  | 56~65 | 120(35.50) |  |  |  |  |
|  | >65 | 119(35.21) |  |  |  |  |
| Educational level (year) | <13 | 62(18.34) | -0.036 | 0.528 | 0.068 | 0.229 |
|  | 13-23 | 274(81.07) |  |  |  |  |
|  | >23 | 2(0.59) |  |  |  |  |
| Family history | First degree relative | 47(13.91) | -0.010 | 0.862 | -0.002 | 0.977 |
|  | Non-first-degree relative | 37(10.95) |  |  |  |  |
|  | Without family history | 253(74.85) |  |  |  |  |
| Age of onset (year) | <60 | 169(50) | 0.053 | 0.347 | -0.016 | 0.774 |
|  | ≥60 | 169(50) |  |  |  |  |
| Age at diagnosis (year) | <60 | 194(57.40) | 0.060 | 0.284 | -0.002 | 0.972 |
|  | ≥60 | 144(42.60) |  |  |  |  |
| Disease duration (year) | <5 | 188(55.62) | -0.079 | 0.163 | -0.050 | 0.372 |
|  | 5-10 | 86(25.44) |  |  |  |  |
|  | >10 | 64(18.93) |  |  |  |  |

**Supplementary Table 2. Follow-up measurements of the participants**

| Scales | T1 | T2 | T3 |
| --- | --- | --- | --- |
| GDS | 2.46±2.72 | 2.57±2.82 | 2.58±2.82 |
| STAI | 65.23±18.56 | 64.78±18.34 | 64.95±18.92 |
| S-anxiety | 32.42±9.96 | 32.32±9.98 | 32.77±9.94 |
| T-anxiety | 32.82±9.65 | 32.46±9.50 | 32.17±10.05 |
| SCOPA-AUT | 10.91±6.23 | 11.45±6.46 | 12.30±7.01 |
| ADL | 90.25±6.75 | 88.62±8.45 | 87.47±8.08 |

Note. GDS, Geriatric Depression Scale; STAI, State-Trait Anxiety Inventory; SCOPA-AUT, Scales for Outcomes in Parkinson’s disease-Autonomic; ADL, activities of daily living

**Supplementary Table 3. Correlation analysis among measurements**

| Variable | GDS1 | STAI1 | SCOPA1 | ADL1 | GDS2 | STAI2 | SCOPA2 | ADL2 | GDS3 | STAI3 | SCOPA3 | ADL3 |
| --- | --- | --- | --- | --- | --- | --- | --- | --- | --- | --- | --- | --- |
| GDS1 | 1 |  |  |  |  |  |  |  |  |  |  |  |
| STAI1 | 0.684** | 1 |  |  |  |  |  |  |  |  |  |  |
| SCOPA1 | 0.393** | 0.367** | 1 |  |  |  |  |  |  |  |  |  |
| ADL1 | -0.206** | -0.164** | -0.179** | 1 |  |  |  |  |  |  |  |  |
| GDS2 | 0.633** | 0.525** | 0.355** | -0.141** | 1 |  |  |  |  |  |  |  |
| STAI2 | 0.558** | 0.715** | 0.297** | -0.111* | 0.652** | 1 |  |  |  |  |  |  |
| SCOPA2 | 0.411** | 0.368** | 0.779** | -0.132* | 0.390** | 0.387** | 1 |  |  |  |  |  |
| ADL2 | -0.282** | -0.213* | -0.247** | 0.577** | -0.259** | -0.261** | -0.245** | 1 |  |  |  |  |
| GDS3 | 0.649** | 0.527** | 0.389** | -0.185** | 0.720** | 0.548** | 0.382** | -0.255** | 1 |  |  |  |
| STAI3 | 0.522** | 0.681** | 0.297** | -0.152** | 0.569** | 0.727** | 0.334** | -0.290** | 0.613** | 1 |  |  |
| SCOPA3 | 0.396** | 0.350** | 0.771** | -0.161** | 0.395** | 0.316** | 0.800** | -0.245** | 0.447** | 0.329** | 1 |  |
| ADL3 | -0.276** | -0.227** | -0.241** | 0.454** | -0.213** | -0.184** | -0.206** | 0.596 | -0.290** | -0.295** | -0.233** | 1 |

Note. *, p<0.05;**, p<0.01.

GDS, Geriatric Depression Scale; STAI, State-Trait Anxiety Inventory; SCOPA-AUT, Scales for Outcomes in Parkinson’s disease-Autonomic; ADL, activities of daily living.
